# Waist-to-height ratio is a simple and practical alternative to waist circumference to diagnose metabolic syndrome in type 2 diabetes

**DOI:** 10.3389/fnut.2022.986090

**Published:** 2022-11-07

**Authors:** Yi-Lin Ma, Chun-Hua Jin, Cui-Chun Zhao, Jiang-Feng Ke, Jun-Wei Wang, Yu-Jie Wang, Jun-Xi Lu, Gao-Zhong Huang, Lian-Xi Li

**Affiliations:** ^1^Department of Endocrinology and Metabolism, Shanghai Sixth People’s Hospital Affiliated to Shanghai Jiao Tong University School of Medicine, Shanghai Clinical Center for Diabetes, Shanghai Key Clinical Center for Metabolic Disease, Shanghai Diabetes Institute, Shanghai Key Laboratory of Diabetes Mellitus, Shanghai, China; ^2^Department of Endocrinology and Metabolism, Shanghai Songjiang District Central Hospital, Songjiang Hospital Affiliated to Shanghai Jiao Tong University School of Medicine (Preparatory Stage), Shanghai, China; ^3^Department of VIP, Shanghai Sixth People’s Hospital Affiliated to Shanghai Jiao Tong University School of Medicine, Shanghai, China

**Keywords:** metabolic syndrome, waist-to-height ratio, type 2 diabetes mellitus, waist circumference, insulin resistance

## Abstract

**Background:**

As an indicator of abdominal obesity, waist circumference (WC) varied with race and gender in diagnosing metabolic syndrome (MetS). Therefore, it is clinically important to find an alternative indicator of abdominal obesity independent of these factors to diagnose MetS. Our aims were to evaluate the association between waist-to-height ratio (WHtR) and MetS and further determine whether WHtR could be used as a simple and practical alternative to WC to diagnose MetS in patients with type 2 diabetes mellitus (T2DM).

**Methods:**

This cross-sectional, real-world study recruited 8488 hospitalized T2DM patients including 3719 women (43.8%) aged from 18 to 94 years and 4769 men (56.2%) aged from 18 to 91 years. A WHtR cut-off of 0.52 was used to diagnose MetS in both men and women T2DM patients based on our previous study. The association of WHtR with MetS in T2DM patients was analyzed by binary logistic regression. The consistency of two diagnostic criteria for MetS according to WC and WHtR was determined by Kappa test.

**Results:**

The prevalence of MetS according to WHtR was 79.4% in women and 68.6% in men T2DM patients, which was very close to the prevalence of MetS according to WC in both women (82.6%) and men (68.3%). The prevalence of MetS diagnosed by WC in both men and women with WHtR ≥ 0.52 was significantly higher than in those with WHtR < 0.52 after adjustment for age and duration of diabetes (89.2 vs. 38.7% for men; 92.8 vs. 57.4% for women; respectively, all *p* < 0.001). Binary logistic regression analysis displayed that after adjusting for confounding factors, WHtR was significantly associated with the presence of MetS in both men and women (men: OR = 4.821, 95% CI: 3.949–5.885; women: OR = 3.096, 95% CI: 2.484–3.860; respectively, all *p* < 0.001). Kappa test revealed that there was an excellent consistency between the diagnosis of MetS based on WC and on WHtR in T2DM patients (men: kappa value = 0.929, 95% CI: 0.918–0.940; women: kappa value = 0.874, 95% CI: 0.854–0.894; total: kappa value = 0.911, 95% CI: 0.901–0.921; respectively, all *p* < 0.001).

**Conclusion:**

WHtR is independently associated with the presence of MetS and can be used as a simple and practical alternative to WC to diagnose MetS regardless of gender in T2DM patients.

## Introduction

Metabolic syndrome (MetS), a cluster of metabolic disorders including central obesity, dyslipidemia, hypertension, and impaired glucose tolerance, refers to a pathological state in which the metabolism of proteins, fats, carbohydrates and other substances in the human body is disturbed (1). There has been a rising incidence of MetS throughout the world. The overall age-standardized prevalence estimates of MetS increased from 13.7 to 31.1% among Chinese adult residents from 2001 to 2017 (2, 3). Furthermore, the prevalence of MetS in patients with glucose regulation disorders was significantly higher than that in those with normal glucose tolerance. According to data from a cohort-study in the Finnish general population, the prevalence of MetS was observed an increasing trend from 12.3% in the subjects with normal glucose tolerance, to 75.1% in those with impaired glucose tolerance and eventually up to 87.1% in patients with diabetes (4).

Metabolic syndrome is presently considered as a prothrombotic and proinflammatory state, and each component of MetS is an independent risk factor for cardiovascular disease and all-cause mortality (5, 6). In a meta-analysis study, Mottillo et al. showed that patients with MetS were at a 2.35-fold increased risk for cardiovascular disease, 1.58-fold for all-cause mortality, 1.99-fold for myocardial infarction, and 2.27-fold for stroke (7). Interestingly, the coexistence of MetS and type 2 diabetes mellitus (T2DM) was independently associated with increased risk of diabetic chronic complications such as chronic kidney disease and cardiovascular diseases (8–10).

Considering the high prevalence and great danger of MetS, it is clinically important to identify MetS early using simple and practical indicators. Different organizations have set different diagnostic criteria of MetS (11–15), but MetS is generally diagnosed in the presence of any 3 of 5 the following medical conditions: abdominal obesity, elevated triglyceride, reduced high-density lipoprotein cholesterol, elevated blood pressure, and elevated fasting glucose (16). Of these medical conditions, abdominal obesity is a necessary condition for the diagnosis of MetS, while the other four conditions remain identical in different criteria for MetS (1, 16). In particular, the diagnostic criteria of MetS by International Diabetes Federation (IDF) was set on the basis of particular anthropometric markers of abdominal obesity, combined with any two additional risk factors (14).

Abdominal obesity, also known as central obesity, is commonly assessed by measurement of waist circumference (WC). Currently, the threshold of WC to define abdominal obesity is quite distinct between different gender and ethnic groups (16). For instance, the recommended WC threshold for abdominal obesity is higher in American and European (≥102 cm in men and ≥88 cm in women) than in Asian (≥90 cm in men and ≥80 cm in women) (16). Thus, it would be of great significance in clinical practice to find a simple and effective alternative to WC to indicate abdominal obesity regardless of gender and race while diagnosing MetS.

In addition to WC, waist-to-height ratio (WHtR) may be a simple and practical anthropometric index to evaluate central obesity. Some studies including our recent study demonstrated that WHtR is superior to WC in assessing central obesity and cardiovascular risk factors (17–20), which indicates that WHtR may be useful to identify MetS. For example, a recent investigation found that WHtR can identify hypertension and dysglycemia well, and can be used as an early warning indicator for MetS in non-overweight/obese Chinese populations (21). Additionally, a strong association between unhealthy WHtR and MetS was also observed in a representative sample of Beijing residents in China (22). Likewise, our recent study also showed that WHtR has a stronger association with cardiovascular risks than WC, and a WHtR cut-off of 0.52 has an excellent diagnostic value for MetS in both male and female T2DM patients, with a good equilibrium in high specificity and sensitivity (20). Therefore, WHtR may be used as a simple and effective alternative to WC to diagnose MetS regardless of sex and race.

To our knowledge, some investigations had found the superiority of applying WHtR to diagnose MetS in Chinese general population, but rarely in diabetic population (23, 24). Furthermore, studies in various ethnic populations have selected different WHtR cut-point for diagnosing MetS (24, 25), and the optimal cut-off value for diagnosing MetS using WHtR is uncertain in Chinese population. Therefore, we conducted the present study in hospitalized T2DM patients with a relatively large sample size to evaluate the association between WHtR and MetS and aimed to determine whether WHtR could be used as a simple and effective alternative to WC to diagnose MetS in T2DM patients.

## Materials and methods

### Study design and subjects

A total of 9621 T2DM patients hospitalized in the Department of Endocrinology and Metabolism of Shanghai Jiao Tong University Affiliated Sixth People’s Hospital from January 2006 to December 2012 were consecutively recruited in this cross-sectional, real-world study. This study was approved by the human ethics committee of Shanghai Jiao Tong University Affiliated Sixth People’s Hospital [approved number: 2018-KY-018(K)], and all participants signed the written informed consent. The present study was conducted in accordance with the Declaration of Helsinki. T2DM patients were diagnosed according to the WHO diagnostic criteria as our previous study (26). The exclusion criteria were as follows: (1) age <18 years old; (2) Patients with diseases affecting WC and WHtR such as malignant tumors; (3) Patients with acute diabetic complications such as diabetic ketoacidosis; (4) Other types of diabetes; (5) Patients lacking information on anthropometric and clinical measurements. Ultimately, 8488 participants were included in this study.

All subjects were interviewed to obtain detailed medical information including age, smoking status, alcohol consumption, duration of diabetes (DD), the history of hypertension, and the history of medication including lipid-lowering drugs (LLDs), insulin or insulin analogs (IIAs) and metformin.

### Physical examination and laboratory tests

Data on weight, height, waist circumference (WC), hip circumference, systolic blood pressure (SBP), and diastolic blood pressure (DBP) were collected as physical measurements. In detail, weight was measured to the nearest half kilogram while wearing light clothing and no shoes. Height, WC and hip circumference were measured to the nearest half centimeter. Blood pressure was measured with a standard mercury sphygmomanometer in a quiet, sedentary state of the subject. Besides, body mass index (BMI), waist-hip ratio (WHR), and waist-to-height ratio (WHtR) were calculated by classical formula as previously described (20, 27), i.e., the weight in kilograms was divided by the square of the height in meters to obtain BMI; WHR was calculated as the WC divided by the hip circumference; and WHtR was calculated as the WC divided by the height. Overnight fasting and 2 h postprandial venous blood samples of the patients were collected for laboratory examinations. Fasting plasma glucose (FPG), 2-h postprandial plasma glucose (2-h PPG) and creatinine (Cr) were measured by oxidase method. Glycosylated hemoglobin A1c (HbA1c) was determined by high performance liquid chromatography. Fasting C-peptide (FCP) and postprandial 2-h postprandial C-peptide (2-h PCP) were performed by electrochemiluminescence. Moreover, the levels of triglyceride (TG), total cholesterol (TC), high-density lipoprotein cholesterol (HDL-C), low-density lipoprotein cholesterol (LDL-C), alanine aminotransferase (ALT), and serum uric acid (SUA) were measured by enzymatic method. 24-h urinary albumin excretion (UAE) and C-reaction protein (CRP) were determined by immunoturbidimetric assay as described in our previous studies (20, 27–29). The calculation of the estimated glomerular filtration rate (eGFR) and the homeostasis model assessment of insulin resistance (HOMA2-IR) were mentioned in our previous studies (20, 27–29).

### Diagnostic criteria

Given that all studied subjects were T2DM patients in the present study, MetS was diagnosed if a patients had any two to four components of MetS including elevated WC or WHtR, elevated TG, reduced HDL-C, and hypertension (16, 20). WHtR ≥ 0.52 was considered as elevated WHtR and elevated WC was defined as WC ≥ 90 cm in men and ≥80 cm in women in the present study (20, 28, 30). Furthermore, the definition of elevated TG, reduced HDL-C, hypertension, the smoking status, and alcohol consumption referred to our previous studies (20, 28, 30).

### Statistical analysis

SPSS 15.0 software was applied for statistical analysis. The normally distributed data were expressed as mean ± standard deviation, and One-way ANOVA with LSD and independent sample *t*-tests were conducted for comparing the differences among different groups. The continuous variables with non-normal distribution were represented by median with interquartile range, and non-parametric test was used to determine the differences between groups. The categorical variables were described as percentages and chi-square test was performed to analyze the differences. The association of WHtR with MetS in T2DM patients was analyzed by binary logistic regression with adjustment for other confounding variables. Kappa test was used to evaluate the consistency of two diagnostic criteria for MetS according to WC and WHtR. *p* < 0.05 was considered as statistically significant difference.

## Results

### Clinical characteristics of the subjects

The clinical characteristics of the T2DM patients are manifested in Table 1. Based on our recent study (16, 20), WHtR of 0.52 was used as a cut-off to divide the subjects into two groups stratified by gender. After adjusting for age, both women and men patients with WHtR ≥ 0.52 had higher prevalence of hypertension, and higher proportion of taking LLDs and metformin compared with those with WHtR < 0.52. Additionally, SBP, DBP, BMI, WC, WHR, FPG, 2h PPG, FCP, 2 h C-P, TG, TC, ALT, Cr, SUA, UAE, and CRP were all significantly increased in the patients with WHtR ≥ 0.52 compared with those with WHtR below 0.52 (all *p* < 0.001). Especially, in women patients, LDL-C levels (*p* = 0.008) and the proportion of LLAs therapy (*p* = 0.030) were also apparently increased in the subjects with WHtR ≥ 0.52. However, HDL-C and eGFR levels were evidently decreased in both women and men patients with WHtR ≥ 0.52 than in those with WHtR < 0.52 (all *p* < 0.05).

**TABLE 1:** The clinical characteristics of the subjects.

	Men				Women			
		
Variables	WHtR < 0.52 (*n* = 1966)	WHtR ≥ 0.52 (*n* = 2803)	*P*-value	*P-value	WHtR < 0.52 (*n* = 1074)	WHtR ≥ 0.52 (*n* = 2645)	*P*-value	*P-value
Age (years)	56 ± 13	59 ± 13	<0.001	−	58 ± 12	63 ± 11	<0.001	−
*DD (months)	72 (12–144)	84 (24–144)	0.023	0.741	96 (36–156)	120 (48–180)	<0.001	0.888
Smoking (*n*, %)	1043 (53.1)	1483 (52.9)	0.922	0.395	21 (2.0)	53 (2.0)	0.924	0.924
Alcohol (*n*, %)	543 (27.6)	876 (31.3)	0.007	0.001	14 (1.3)	25 (0.9)	0.331	0.519
Hypertension (*n*, %)	752 (38.3)	1654 (59.0)	<0.001	<0.001	461 (42.9)	1724 (65.2)	<0.001	<0.001
LLD (*n*, %)	608 (30.9)	1292 (46.1)	<0.001	<0.001	358 (33.3)	1174 (44.4)	<0.001	<0.001
Metformin (*n*, %)	976 (49.6)	1796 (64.1)	<0.001	<0.001	563 (52.4)	1704 (64.4)	<0.001	<0.001
IIAs (*n*, %)	1403 (71.4)	1969 (70.2)	0.404	0.404	739 (68.8)	1914 (72.4)	0.030	0.030
SBP (mmHg)	128 ± 16	132 ± 17	<0.001	<0.001	129 ± 17	136 ± 17	<0.001	<0.001
DBP (mmHg)	79 ± 9	81 ± 10	<0.001	<0.001	78 ± 9	80 ± 9	<0.001	<0.001
BMI (kg/m2)	22.7 ± 2.4	26.7 ± 2.8	<0.001	<0.001	21.8 ± 2.5	26.3 ± 3.3	<0.001	<0.001
WC (CM)	82.3 ± 6.3	97.2 ± 7.5	<0.001	<0.001	76.7 ± 5.6	92.9 ± 8.2	<0.001	<0.001
WHR	0.89 ± 0.05	0.96 ± 0.05	<0.001	<0.001	0.85 ± 0.05	0.93 ± 0.06	<0.001	<0.001
*FPG (mmol/l)	7.59 (5.99–9.77)	7.91 (6.37–10.03)	<0.001	<0.001	7.27 (5.88–9.22)	7.82 (6.32–9.84)	<0.001	<0.001
*2-h PPG (mmol/l)	12.78 (9.35–16.45)	13.62 (10.70–16.80)	<0.001	<0.001	12.39 (9.19–15.80)	13.60 (10.46–17.10)	<0.001	<0.001
HbA1c (%)	9.29 ± 2.55	8.98 ± 2.16	<0.001	<0.001	8.71 ± 2.36	8.84 ± 2.08	0.105	0.033
*FCP (ng/mL)	1.42 (0.84–2.05)	2.02 (1.31–2.81)	<0.001	<0.001	1.49 (0.95–2.09)	1.95 (1.29–2.78)	<0.001	<0.001
*2-h PCP (ng/mL)	3.07 (1.63–5.07)	4.34 (2.67–6.18)	<0.001	<0.001	3.46 (1.93–5.45)	4.30 (2.61–6.28)	<0.001	<0.001
*TG (mmol/l)	1.15 (0.80–1.67)	1.61 (1.13–2.40)	<0.001	<0.001	1.19 (0.83–1.70)	1.59 (1.13–2.29)	<0.001	<0.001
TC (mmol/l)	4.54 ± 1.18	4.66 ± 1.17	0.001	<0.001	4.86 ± 1.12	5.00 ± 1.16	0.001	<0.001
HDL-C (mmol/l)	1.13 ± 0.31	1.00 ± 0.24	<0.001	<0.001	1.30 ± 0.39	1.17 ± 0.30	<0.001	<0.001
LDL-C (mmol/l)	2.94 ± 0.95	2.95 ± 0.91	0.639	0.405	3.08 ± 1.01	3.16 ± 0.95	0.015	0.008
*ALT (u/l)	18 (13–28)	22 (16–35)	<0.001	<0.001	16 (12–24)	19 (13–30)	<0.001	<0.001
*Cr (μmol/l)	73 (64–84)	75 (65–88)	<0.001	<0.001	54 (47–63)	57 (49–67)	<0.001	<0.001
*SUA (μmol/l)	309 (261–368)	348 (294–407)	<0.001	<0.001	265 (221–316)	299 (249–360)	<0.001	<0.001
*UAE (mg/24 h)	9.74 (6.07–22.72)	14.13 (7.43–46.42)	<0.001	<0.001	9.08 (5.88–18.59)	13.66 (7.86–40.07)	<0.001	<0.001
*eGFR (ml/min/1.73 m^2^)	109 (91–130)	104 (85–125)	<0.001	<0.001	124 (102–147)	114 (92–139)	<0.001	<0.001
*CRP (mg/l)	0.75 (0.33–1.86)	1.27 (0.60–3.11)	<0.001	<0.001	0.70 (0.32–1.69)	1.50 (0.72–3.47)	<0.001	<0.001

Values are expressed as the mean ± SD, or median with interquartile range, or percentages.

*P*-value: the *p*-values were not adjusted for age for the trend.

*P-value: the *p-values were adjusted for age for the trend.

### Comparisons of metabolic syndrome prevalence

**FIGURE 1:**
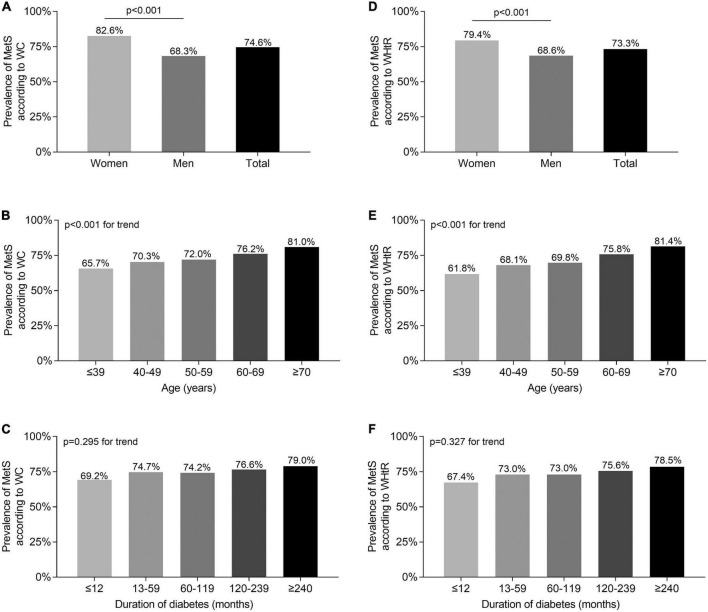
Comparisons of MetS prevalence diagnosed according to WC and WHtR in T2DM patients. **(A)** Comparison of the prevalence of MetS diagnosed by WC stratified by sex after adjusting for age and DD. **(B)** Comparison of the prevalence of MetS diagnosed by WC stratified by age after adjusting for sex and DD. **(C)** Comparison of the prevalence of MetS diagnosed by WC stratified by DD after adjusting for sex and age. **(D)** Comparison of the prevalence of MetS diagnosed by WHtR stratified by sex after adjusting for age and DD. **(E)** Comparison of the prevalence of MetS diagnosed by WHtR stratified by age after adjusting for sex and DD. **(F)** Comparison of the prevalence of MetS diagnosed by WHtR stratified by DD after adjusting for sex and age.

The comparisons of the prevalence of MetS diagnosed by either WC or WHtR stratified by gender, age, and DD are demonstrated in Figure 1. The prevalence of MetS according to WC was 82.6% in women and 68.3% in men T2DM patients (*p* < 0.001) (Figure 1A), which was very close to the prevalence of MetS according to WHtR in women (79.4%) and men (68.6%) (all *p* < 0.001) (Figure 1D). Moreover, with the increase of age, there was a similarly increased trend in the prevalence of MetS according to WC (65.7, 70.3, 72.0, 76.2, and 81.0% for different age groups, respectively, *p* < 0.001) (Figure 1B) and WHtR (61.8, 68.1, 69.8, 75.8, and 81.4% for different age groups, respectively, *p* < 0.001) (Figure 1E). However, there was no significant difference in the MetS prevalence among different DD groups, whether the diagnosis of MetS was based on WC (69.2, 74.7, 74.2, 76.6, and 79.0% for different DD groups, respectively, *p* = 0.295 for trend) (Figure 1C) or WHtR (67.4, 73.0, 73.0, 75.6, and 78.5% for different DD groups, respectively, *p* = 0.327 for trend) (Figure 1F).

### Comparisons of the metabolic syndrome components

**FIGURE 2:**
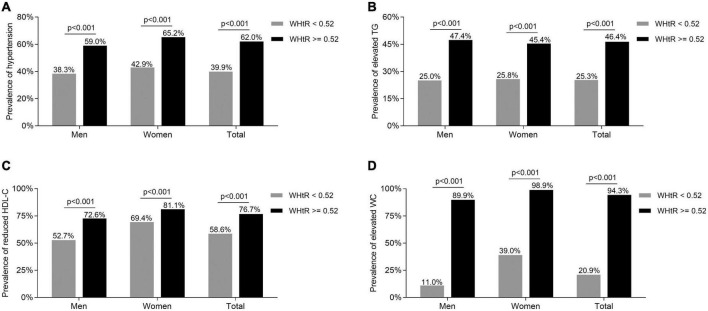
Comparisons of the MetS components between the T2DM subjects with WHtR < 0.52 and ≥0.52. **(A)** Comparison of the prevalence of hypertension stratified by sex between the subjects with WHtR < 0.52 and ≥0.52 after adjusting for age and DD. **(B)** Comparison of the prevalence of elevated TG stratified by sex between the subjects with WHtR < 0.52 and ≥0.52 after adjusting for age and DD. **(C)** Comparison of the prevalence of reduced HDL-C stratified by sex between the subjects with WHtR < 0.52 and ≥0.52 after adjusting for age and DD. **(D)** Comparison of the prevalence of elevated WC stratified by sex between subjects with WHtR < 0.52 and ≥0.52 after adjusting for age and DD.

The comparisons of the MetS components including hypertension, elevated TG, reduced HDL-C, and elevated WC between the subjects with WHtR ≥ 0.52 and <0.52 are presented in Figure 2. After adjusting for age and DD, the prevalence of hypertension, elevated TG, reduced HDL-C, and elevated WC were all obviously higher in the subjects with WHtR ≥ 0.52 than in those with WHtR < 0.52 in both men and women (hypertension: 59.0 vs. 38.3% for men, 65.2 vs. 42.9% for women, respectively; elevated TG: 47.4 vs. 25.0% for men, 45.4 vs. 25.8% for women, respectively; reduced HDL-C: 72.6 vs. 52.7% for men, 81.1 vs. 69.4% for women, respectively; elevated WC: 89.9 vs. 11.0% for men, 98.9 vs. 39.0% for women, respectively; all *p* < 0.001) (Figures 2A–D).

### Comparisons of the metabolic syndrome prevalence diagnosed by waist circumference between the subjects with waist-to-height ratio < 0.52 and ≥0.52

**FIGURE 3:**
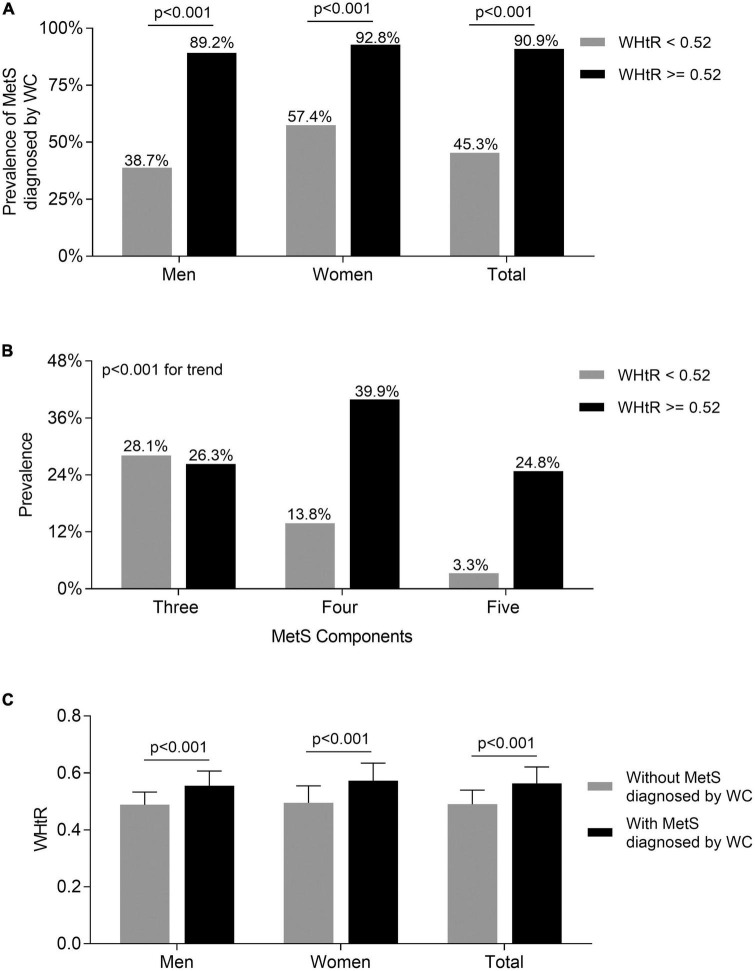
Comparisons of the prevalence of MetS diagnosed by WC between the subjects with WHtR < 0.52 and ≥0.52. **(A)** Comparison of the prevalence of MetS diagnosed by WC stratified by sex between the subjects WHtR < 0.52 and ≥0.52 after adjusting for age and DD. **(B)** Comparison of the number of MetS components between the subjects with WHtR < 0.52 and ≥0.52. **(C)** Comparison of the values of WHtR stratified by sex between the subjects with and without MetS diagnosed by WC after adjusting for age and DD.

As Figure 3 shows, the prevalence of MetS diagnosed by WC in both men and women with WHtR ≥ 0.52 was significantly higher than in those with WHtR < 0.52 after adjustment for age and DD (89.2 vs. 38.7% for men, 92.8 vs. 57.4% for women, respectively; all *p* < 0.001) (Figure 3A). Further, there was a significantly increased trend in the prevalence of more MetS components when patients’ WHtR ≥ 0.52 compared with those with WHtR below 0.52 (three MetS components: 26.3 vs. 28.1%; four MetS components: 39.9 vs. 13.8%; five MetS components: 24.8 vs. 3.3%; *p* < 0.001 for trend) (Figure 3B). Additionally, the T2DM patients with MetS diagnosed by WC had an obviously higher WHtR than those without MetS in both men and women (0.55 ± 0.05 vs. 0.49 ± 0.04 for men; 0.57 ± 0.06 vs. 0.49 ± 0.06 for women; 0.56 ± 0.06 vs. 0.49 ± 0.05 for total, respectively; all *p* < 0.001) (Figure 3C).

### Correlation of waist-to-height ratio with insulin resistance

**FIGURE 4:**
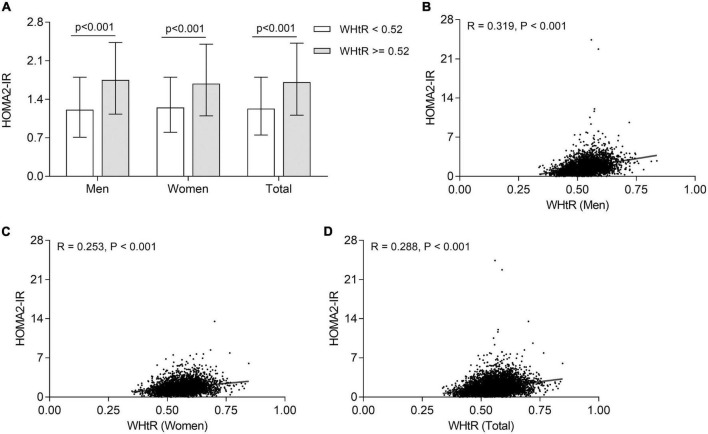
Association of WHtR with insulin resistance in T2DM patients. **(A)** Comparison of HOMA2-IR stratified by sex between the subjects with WHtR < 0.52 and ≥0.52 after adjustment for age and DD. **(B)** Correlation of WHtR with HOMA2-IR in men T2DM patients. **(C)** Correlation of WHtR with HOMA2-IR in women T2DM patients. **(D)** Correlation of WHtR with HOMA2-IR in all T2DM patients.

The comparison of insulin resistance between the T2DM patients with WHtR ≥ 0.52 and <0.52 is displayed in Figure 4A. Compared with those with WHtR < 0.52, after adjustment for age and DD, T2DM patients with WHtR ≥ 0.52 had an enhanced insulin resistance with a higher value of HOMA2-IR in both men and women [1.75 (1.13–2.43) vs. 1.21 (0.71–1.8) for men; 1.69 (1.10–2.40) vs. 1.25 (0.80–1.80) for women; 1.71 (1.11–2.42) vs. 1.23 (0.75–1.8) for total, respectively; all *p* < 0.001]. Furthermore, after controlling for age and DD, partial correlation analysis showed that WHtR was positively correlated with insulin resistance in both men (*R* = 0.319, *p* < 0.001) (Figure 4B), women (*R* = 0.253, *p* < 0.001) (Figure 4C), and all T2DM patients (*R* = 0.288, *p* < 0.001) (Figure 4D).

### Association of waist-to-height ratio with metabolic syndrome

Table 2 shows the association of WHtR with MetS in T2DM patients stratified by gender. Binary logistic regression analysis displayed that after adjusting for confounding variables including age, DD, smoking, alcohol drinking (model 1), WHtR was significantly associated with the presence of MetS in both men (OR = 7.414, 95% CI: 6.540–8.405, *p* < 0.001) and women (OR = 4.772, 95% CI: 4.174–5.457, *p* < 0.001). After further controlling for the use of LLDs, IIAs, and metformin (model 2), SBP, DBP, and BMI (model 3), ALT, LDL-C, TC, eGFR, SUA, HbA1c, FPG, 2h PPG, FCP, 2h PCP, UAE, and CRP (model 4), WHtR was still independently correlated with the presence of MetS in both men (model 2: OR = 7.883, 95% CI: 6.864–9.054; model 3: OR = 5.127, 95% CI: 4.310–6.099; model 4: OR = 4.821, 95% CI: 3.949–5.885; respectively, all *p* < 0.001) and women (model 2: OR = 5.081, 95% CI: 4.413–5.850; model 3: OR = 2.926, 95% CI: 2.437–3.514; model 4: OR = 3.096, 95% CI: 2.484–3.860; respectively, all *p* < 0.001).

**TABLE 2:** The association of WHtR with MetS in T2DM patients.

	Men		Women		Total	
			
	OR (95% CI)	*P*-value	OR (95% CI)	*P*-value	OR (95% CI)	*P*-value
Model 1	7.414 (6.540–8.405)	<0.001	4.772 (4.174–5.457)	<0.001	6.191 (5.652–6.782)	<0.001
Model 2	7.883 (6.864–9.054)	<0.001	5.081 (4.413–5.850)	<0.001	6.459 (5.848–7.133)	<0.001
Model 3	5.127 (4.310–6.099)	<0.001	2.926 (2.437–3.514)	<0.001	4.060 (3.576–4.610)	<0.001
Model 4	4.821 (3.949–5.885)	<0.001	3.096 (2.484–3.860)	<0.001	3.903 (3.368–4.523)	<0.001

Model 1: adjusted for age, DD, smoking, alcohol drinking (gender adjustment for total subjects). Model 2: further adjusted for the use of LLDs, insulin or insulin analogs, and metformin (gender adjustment for total subjects). Model 3: further adjusted for SBP, DBP, and BMI (gender adjustment for total subjects). Model 4: further adjusted for ALT, LDL-C, TC, eGFR, Cr, eGFR, SUA, HbA1c, FPG, 2-h PPG, FCP, 2-h PCP, UAE, and CRP (gender adjustment for total subjects).

### The consistency of diagnosis of metabolic syndrome according to waist circumference and waist-to-height ratio

As shows in Table 3, there was an excellent consistency between the diagnosis of MetS according to WC and WHtR in both men (kappa value = 0.929, 95% CI: 0.918–0.940, *p* < 0.001) and women T2DM patients (kappa value = 0.874, 95% CI: 0.854–0.894, *p* < 0.001).

**TABLE 3:** The consistency of diagnosis of MetS according to WC and WHtR.

	Kappa values	95% CI	*P*-value
Men	0.929	0.918–0.940	<0.001
Women	0.874	0.854–0.894	<0.001
Total	0.911	0.901–0.921	<0.001

## Discussion

The superiority of applying WHtR to diagnose MetS in T2DM subjects has not been clearly confirmed so far, and the optimal cut-off value of WHtR for diagnosing MetS remains uncertain in the Chinese population. Therefore, we designed this large sample-size, real-world study to evaluate the association between WHtR and MetS, and further examine whether WHtR could be used as a simple and effective alternative to WC to diagnose MetS in T2DM. In fact, we observed a significantly positive association between WHtR and the presence of MetS in both men and women T2DM patients. Additionally, there was an excellent agreement between the diagnosis of MetS by WC and by WHtR ≥ 0.52 in T2DM patients.

Metabolic syndrome is a cluster of cardiovascular risk factors such as hypertension, dyslipidemia, and diabetes, and is firstly defined by the World Health Organization (WHO) in 1998 (11). Since that time, several other diagnostic criteria of MetS have been respectively released by the National Cholesterol Education Program Adult Treatment Panel III (NCEP-ATP III), the American Association of Clinical Endocrinologists (AACE), the International Diabetes Federation (IDF), and the American Heart Association/National Heart, Lung, and Blood Institute (AHA/NHLBI) (12–15). The definition of MetS is different in different diagnostic criteria, but abdominal obesity is an indispensable component of MetS. It was reported that adiponectin and inflammatory cytokines secreted by excessive visceral adipose tissue promote the generation of insulin resistance, a key factor associated with a set of metabolic abnormalities in MetS (31). Therefore, abdominal obesity plays a crucial role in the pathogenesis of MetS and is a key feature in diagnosing MetS.

Currently, WC is usually selected as an anthropometric indicator to evaluate visceral fat accumulation and thus to determine the presence of abdominal obesity. Whereas the cut-point of WC to diagnose abdominal obesity varied among subjects with different sex and race, which have greatly limited its applicability in assessing MetS. For example, the reference threshold of WC for distinguishing abdominal obesity is 102 cm in men and 88 cm in women for Canadians, Americans, and Europeans, 90 cm in men and 80 cm in women for Asians, 94 cm in men and 80 cm in women for Mediterranean and Africans (16).

Contrarily, WHtR is a relatively constant indicator of abdominal obesity across different sex, age and ethnic groups with little variation compared with WC, which reflects the possible uniqueness of WHtR in predicting abdominal obesity and MetS (32, 33). WHtR is generally regarded as an excellent body fat discriminator in both sexes (34). Nevill and colleagues reported that WHtR retained a stronger association with subcutaneous central obesity than absolute WC (35). Furthermore, WHtR was a better predictor to detect general and central obesity compared with WC among children and adolescents of different ages and genders (36). Additionally, several studies suggested that WHtR presented significantly better predictive and discriminatory power than WC for diabetes, dyslipidemia, hypertension, and cardiovascular disease in ethnically and racially diverse populations and in both sexes (18, 37). Therefore, by contrast with WC, WHtR can better assess abdominal obesity and other metabolic disorders across different ethnicity and sex.

In addition to as a useful indicator of abdominal obesity, WHtR was also applied to evaluate the risk of other metabolic disorders such as diabetes, hypertension, and dyslipidemia, which belong to MetS components. An increasing number of studies have demonstrated the strong association between WHtR and the development of cardiovascular diseases and MetS components (38–41). For example, a recent follow-up study found that WHtR was a useful and accurate parameter to predict the occurrence of hypertension in T2DM patients (38). Additionally, Cao et al. reported that WHtR ≥ 0.5 was markedly linked with higher risk of dyslipidemia compared to WHtR < 0.5 in Chinese Adults (39). Aligned with them, we also found that the prevalence of multiple metabolic disorders such as hypertension, elevated TG, and reduced HDL-C were obviously higher in both men and women T2DM subjects with WHtR ≥ 0.52 than in those with WHtR < 0.52. T2DM patients with four or five MetS components were more likely to be those with WHtR ≥ 0.52. Furthermore, fully adjusted regression analyses also revealed that WHtR was independently associated with the development of MetS in T2DM patients, which was consistent with recent studies by Guo et al. and Savva et al. (42, 43).

More importantly, based on our recent study (20), we chose a WHtR of 0.52 as the appropriate cut-off point for diagnosing MetS in Chinese adults with T2DM patients in the present study, which was same to the results reported by two previous studies (23, 44), and showed an excellent diagnostic value for MetS in both men (70.4% sensitivity and 84.7% specificity) and women (80.1% sensitivity and 83.4% specificity) T2DM patients according to ROC curve analysis based on our previous study (20). Furthermore, our study manifested that the prevalence of MetS diagnosed by WHtR was highly consistent with that determined by WC in different age, sex, DD groups. Additionally, the Kappa test revealed an excellent agreement between the prevalence of MetS diagnosed by WC and WHtR in T2DM patients. Consequently, our findings provided a further possibility for WHtR to replace WC as an indicator of abdominal obesity to diagnose MetS in T2DM subjects regardless of sex.

Contrary to our choice, Pan el al suggested that the optimal cut-off levels of WHtR for predicting two or more non-adipose components of MetS including hypertension, dyslipidemia, and hyperglycemia were 0.50 in men and 0.51 in women in a southeast rural Chinese population after comparing the MetS under different definitions (45), but this WHtR cut-off might only apply to the selected subjects aged 40 years and older. Likewise, Shao et al. argued that the optimal cut-off points for screening obesity in MetS subjects were approximately 0.50 in both genders in 2947 Chinese adults (46). In contrast, our present study was based on T2DM participants with more severe abdominal obesity than general population, which might result in a larger WHtR cut-point with 0.52 in T2DM patients than 0.50 in general residents. However, the large sample size of 8488 ensures the reliability of our findings. Apart from subtle differences in the population, there might be ethnic differences in the diagnosis of MetS by WHtR. A survey of Ethiopian adults showed the WHtR cut-off scores for detecting MetS ranged from 0.47 to 0.53 in men and from 0.47 to 0.56 in women due to adopting different MetS components to diagnose MetS (25). In the Polish population, the appropriate cut-offs for MetS identification by WHtR were 0.556 in men and 0.535 in women (24). Although there might be population and racial differences in the diagnosis of MetS using WHtR, these differences are much smaller than that using WC, which suggests the possibility that WHtR may substitute for WC in diagnosing MetS. However, large sample studies in different populations and races are needed to clarify the optimal cut-point for WHtR in identifying MetS.

Insulin resistance is the main reason that WHtR is closely related to MetS and can be used as an indicator of MetS in T2DM subjects. Insulin resistance was often regarded as the hallmark feature and core mechanism of the MetS (47, 48). Therefore, the elevation of insulin levels in MetS precedes other metabolic disorders and MetS arises from insulin resistance (49). Hyperinsulinemia markedly activates the sympathetic nervous system and renal sodium reabsorption, thereby inducing the development of hypertension (48). In addition, insulin resistance increases hepatic very low-density lipoprotein (VLDL) production and decreases HDL production, thereby increasing serum TG levels and decreasing serum HDL levels (48). Besides, the presence of hyperinsulinemia and hepatic insulin resistance accelerated liver endogenous glucose production, suppressed glucose uptake in skeletal muscle and further drove the development of hyperglycemia (50). Therefore, hypertension, dyslipidemia, and hyperglycemia caused by insulin resistance combining with central obesity constitute the components of MetS. Correspondingly, our study demonstrated that WHtR was positively correlated with insulin resistance, and T2DM patients with WHtR ≥ 0.52 had significantly enhanced insulin resistance with a higher value of HOMA2-IR compared with those with WHtR < 0.52. In line with our findings, a cross-sectional study found that HOMA-IR values were significantly higher in polycystic ovarian syndrome patients with WHtR > 0.5 than in those with WHtR ≤ 0.5 (51). Moreover, Lechner et al. reported that the prevalence of insulin resistance defined by the Matsuda index obviously increased with elevated WHtR, and the predictive ability of WHtR for insulin resistance was highly accurate, especially in T2DM population (52). Thus, it is feasible and reasonable to use WHtR instead of WC to diagnose MetS given that WHtR clearly indicates and closely correlates with insulin resistance.

Our study has practical implications. MetS poses one of the major challenges for global and national public health agencies as an accumulation of multiple health risk factors that are associated with increased risks of developing cardiovascular diseases, non-alcoholic fatty liver disease and all-cause mortality (7, 53). Our present study strongly suggests WHtR ≥ 0.52 as an early warning of health risk for predicting MetS in Chinese T2DM patients. In addition, this universal cut-off value for WHtR eliminates the need for age-, gender-, and race-specific thresholds for MetS, enabling people to monitor their own physical health risks individually and conveniently. As an indicator to assess the risk of MetS, the clinical application and promotion of the WHtR contribute to the early adoption of preventive strategies to reduce the risk of metabolic-related diseases and improve the overall health status of the population.

However, there are also some limitations in our study. Firstly, the recruited subjects in the present study were from T2DM population, thus our findings may not be fully applicable to other populations. Also, the WHtR value for predicting MetS is racial differences, it is necessary to find an optimal cut-point for WHtR in diagnosing MetS in different races in future studies. Secondly, some other factors such as the use of IIAs and metformin may affect WC and WHtR, but we eliminated the influence of these factors as much as possible in analyses. Thirdly, the subjects in this study mainly came from single-center hospitalized patients, and thus their characteristics might not comprehensively reflect the overall health status of T2DM population. Thus, the multi-center investigation is needed in subsequent related studies.

In conclusion, WHtR is closely and independently associated with the presence of MetS in both men and women T2DM subjects. WHtR ≥ 0.52 may be used as a simple and practical alternative to WC to diagnose MetS regardless of gender in Chinese T2DM patients.

## Data availability statement

The original contributions presented in this study are included in the article/supplementary material, further inquiries can be directed to the corresponding authors.

## Ethics statement

The studies involving human participants were reviewed and approved by the Human Ethics Committee of Shanghai Jiao Tong University Affiliated Sixth People’s Hospital. The patients/participants provided their written informed consent to participate in this study.

## Author contributions

G-ZH and L-XL designed the study, reviewed, and edited the manuscript. Y-LM, J-WW, J-FK, Y-JW, and J-XL collected the samples and clinical data. Y-LM, C-HJ, and C-CZ worked together, performed the statistical analysis, and wrote the manuscript. All authors revised the manuscript and approved the final manuscript.
